# Optimization of Ultrasound-Assisted Extraction, HPLC and UHPLC-ESI-Q-TOF-MS/MS Analysis of Main Macamides and Macaenes from Maca (Cultivars of *Lepidium meyenii* Walp)

**DOI:** 10.3390/molecules22122196

**Published:** 2017-12-10

**Authors:** Shu-Xiao Chen, Ke-Ke Li, Duoji Pubu, Si-Ping Jiang, Bin Chen, Li-Rong Chen, Zhen Yang, Chao Ma, Xiao-Jie Gong

**Affiliations:** 1 College of Environmental and Chemical Engineering, Dalian University, Dalian 116622, China; 18340862946@163.com (S.-X.C.); chenlirong@dlu.edu.cn (L.-R.C.); 2 College of Medical, Dalian University, Dalian 116622, China; 3 Tibet Plateau Institute of Biology, Lhasa 850001, China; puduo@126.com (D.P.); tpibjiangsp@126.com (S.-P.J.); wvoo@163.com (C.M.); 4 School of Marine Sciences, Sun Yat-sen University, Zhuhai 519082, China; chenyishan@126.com; 5 HPV Immunology Lab, Leidos Biomedical Research Inc., Frederick National Laboratory for Cancer Research, Frederick, MD 21702, USA; zhenyang2@yahoo.com

**Keywords:** cultivated maca, macamides, ultrassound-assisted extraction, response surface methodology, optimization, UHPLC-ESI-Q-TOF-MS/MS

## Abstract

Ultrasound-assisted extraction (UAE), using petroleum ether as the solvent, was systematically applied to extract main macamides and macaenes from Maca hypocotyls. Extraction yield was related with four variables, including ratio of solution to solid, extraction temperature, extraction time, and extraction power. On the basis of response surface methodology (RSM), the optimal conditions were determined to be the ratio of solution to solid as 10:1 (mL/g), the extraction temperature of 40 °C, the extraction time of 30 min, and the extraction power of 200 W. Based on the optimal extraction method of UAE, the total contents of ten main macamides and two main macaenes of Maca cultivated in twenty different areas of Tibet were analyzed by HPLC and UHPLC-ESI-Q-TOF-MS/MS. This study indicated that UAE was able to effectively extract macamides alkaloids from Maca hypocotyls. Quantitative analysis showed that geographical origins, not ecotypes, played a more important role on the accumulation of active macamides in Maca.

## 1. Introduction

Maca (*Lepidium meyenii* Walp), a plant that grows in above 4000 meters of altitude in Peru’s Central Andes, contains hypocotyls that have been used as food and in traditional medicine for centuries. It has also been cultivated in Tibet, Yunnan and Xinjiang of China over the past decade. Dried Maca hypocotyls contain several classes of secondary metabolites of interest including alkaloids, amino acids, glucosinolates, polysaccharides, fatty acids and macamides [1]. Among them, macamides, a group of non-polar, long-chain fatty acid *N*-benzylamides compounds, were identified as the characteristic constituents while contributing to the major efficacies in Maca such as anti-fatigue [2], exciting central nervous system (anti-depressant, anti-anxiety and analgesic) [3,4,5], neuroprotective [6,7], anti-osteoporosis [8], enhancing sexual function and improving fertility [9,10,11]. It was observed that recovery of macamides from Maca hypocotyls has economical benefits for both the both food and pharmaceutical industry.

The studies reported up to date describe designs for the extraction of secondary metabolites in Maca. These studies include the optimization of temperature, liquid-to-solid ratio, time and ethanol concentration for glucosinolate and phenolic compound extraction from dried Maca hypocotyls using response surface methodology (RSM) [12]; the extraction of water-soluble polysaccharides from Yunnan Maca aqueous extract by the Sevag method [13]; the optimization of glucosinolate extraction from Maca by combined ultrasonic and microwave extraction with RSM [14]. As for the macamides, most of the reports focus on phytochemistry studies of their separation and analysis [1,7,15,16], and very few researchers have previously reported an extraction methodology to obtain macamides from Maca hypocotyls.

Ultrasound-assisted extraction (UAE) has been reported as a useful extraction technique to recover, identify, and quantify alkaloid components from medicinal and food materials [17,18,19,20]. UAE has received considerable attention due to its positive influence on heat and mass transfer and hence it is considered a green technology [21,22,23]. It permits an efficient extraction of bioactive molecules in a shorter time and at lower temperatures than traditional methods such as solvent refluxing and maceration. However, to date there is lack of information regarding the optimization of conditions for the UAE of macamides alkaloids compounds extracted from Maca hypocotyls. The aim of this study was therefore to investigate the effect of different extraction parameters (ratio of solution to solid, temperature, ultrasound time and power) on the contents of the main macamides and macaenes by applying RSM in order to optimize these extraction parameters. The obtained eleven macamides and two macaenes compounds were determined by ultra-high performance liquid chromatography coupled with electrospray ionization quadrupole time-of-flight tandem mass spectrometry (UHPLC-EIS-Q-TOF-MS/MS). The total contents of macamides of the cultivated Maca (three ecotypes: black, yellow and purple) from Tibet and the differences between them were also discussed.

## 2. Results and Discussion

### 2.1. Single Factor Test

**Figure 1:**
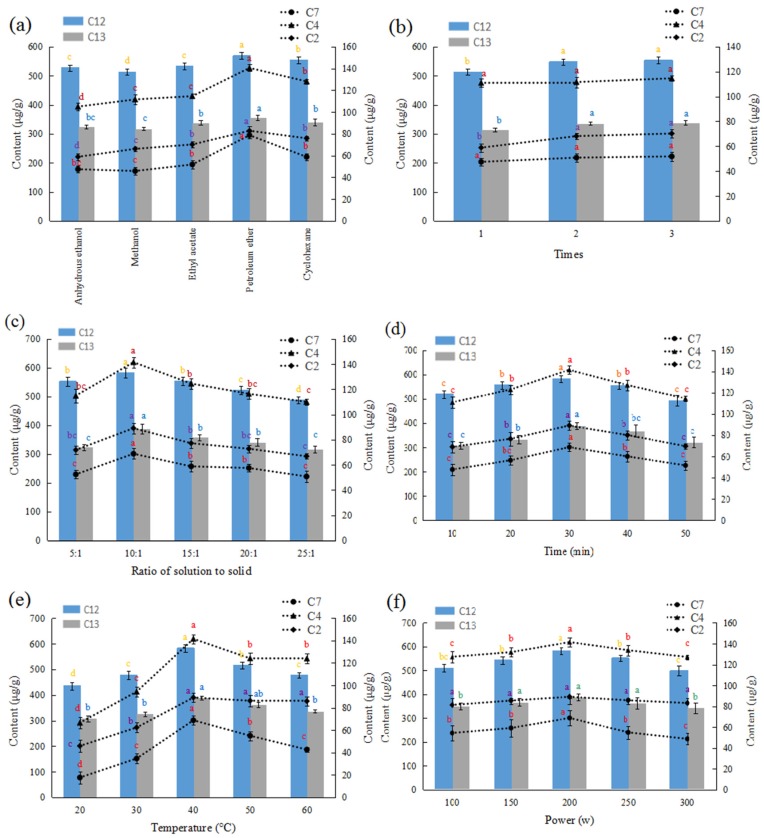
Effect of extraction solvent (**a**), extraction frequency (**b**), ratio of solution to solid (**c**), extraction time (**d**), extraction temperature (**e**), extraction power (**f**), on the yields of macamides and macaenes in single factor experiments (different letters stand for significant difference at 5% level; three macamides, C2: *N*-benzyl-(9*Z*,12*Z*,15*Z*)-octadecatrienamide, C4: *N*-benzyl-(9*Z*,12*Z*)-octadecadienamide, C7: *N*-benzylhexadecanamide and two macaenes, C12: 9*E*,12*E*,15*E*-octadecadienoic acid, C13: 9*E*,12*E*-octadecadienoic acid are shown).

Macamides and macaenes are a group of non-polar compounds, so compared with the extraction efficiency of other solvents, petroleum ether could achieve a better extraction for the five typical compounds (C2, C4, C7, C12, and C13) which are most abundant components in Maca, as shown in Figure 1a, with significant differences at *P* < 0.05 in their extraction yield. Thus, petroleum ether was selected as the extraction solvent to extract macamides and macaenes. As for the effect of extraction frequency on the extraction yield, the results are shown in Figure 1b. In this study, two and three extractions showed better results in extracting macamides and macaenes. The double and triple extractions did not differ significantly, so an appropriate extraction frequency was set as twice. Thus, the ratio of solution to solid (Figure 1c), extraction time (Figure 1d), extraction temperature (Figure 1e), and extraction power (Figure 1f) were regard as the main variables, which ranged from 5:1 to 25:1 (mL/g), 10 to 50 min, 20 to 60 °C, and 100 to 300 W, respectively.

### 2.2. Statistical Analysis and Model Fitting using RSM

Twenty-nine experiments were designed and a Box-Behnken design (BBD) of RSM was carried out to optimize the UAE conditions. The results are listed in Table 1.

According to a regression analysis of the experimental data, the extraction efficiency could be explained by the following polynomial equations (Equation (1)):

(1) $$Y=1169.18-29.75X_{1}-24.32X_{2}-5.16X_{3}+11.48X_{4}+3.64X_{1}X_{2}+14.70X_{1}X_{3}-\;37.48X_{1}X_{4}+27.63X_{2}X_{3}-8.15X_{2}X_{4}-12.16X_{3}X_{4}-64.25X_{1}^{2}-71.05X_{2}^{2}-40.42X_{3}^{2}-\;87.36X_{4}^{2}$$

where *Y* represents the yield of three macamides and two macaenes; *X*_1_, *X*_2_, *X*_3_ and *X*_4_ are the coded variables for the ratio of solution to solid, extraction temperature, extraction time, and extraction power, respectively.

As shown in Table 2, the *F*-value and *P*-value of the model were 22.28 and < 0.0001, respectively, which suggested the model was significant. The coefficients *X*_1_, *X*_1_^2^, *X*_2_^2^, *X*_3_^2^, and *X*_4_^2^ showed significant differences at *P* < 0.0001, *X*_2_ and *X*_1_*X*_4_ showed significant differences at *P* < 0.01, *X*_2_*X*_3_ showed significant differences at *P* < 0.05, while the other coefficients were insignificant (*P* > 0.05). In addition, *P*-value of the lack of fit was 0.1160, which implied the lack of fit was insignificant compared to the pure error. The value of determination coefficient (*R*^2^ = 0.9571) for this model was close to 1, indicating a high degree of correlation between the observed and predicted values. The value of adjusted determination coefficients (Adjust *R*^2^) was also close to 1, which indicated the experimental values could be significantly predicted by the model.

**Figure 2:**
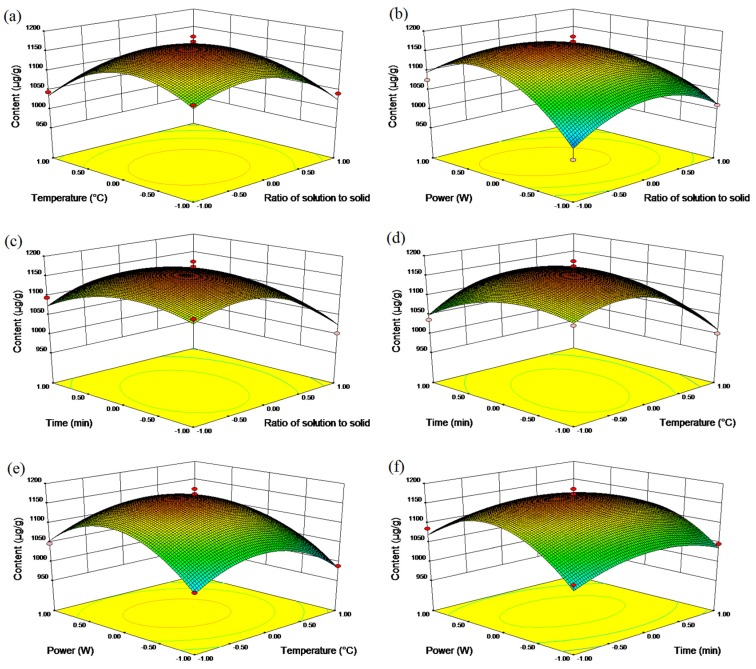
The 3D response surface of macamides and macaenes affected by ratio of solution to solid, extraction temperature, extraction time, and extraction power.

Three-dimensional (3D) response surface, as an essential part of regression equation, could vividly expound the interactions between two variables and determine their optimal levels (Figure 2). The detailed descriptions were as follows: (a) the strong interaction between *X*_1_ (ratio of solution to solid) and *X*_2_ (temperature) was investigated while other variables were held constant. When *X*_1_ was fixed, the contents of three macamides and two macaenes increased continuously, and reached the maximum when *X*_1_ and *X*_2_ became approximately 10:1 and 40 °C, respectively. Beyond this level, the yield reduced with the increase of *X*_1_ and *X*_2_. The same variation of yield caused by *X*_2_ was also observed. Hence, the interactive effect of *X*_1_ and *X*_2_ was remarkable; (b) the contents of three macamides and two macaenes increased linearly with the increase of *X*_4_ (power) at a fixed *X*_1_ (ratio of solution to solid), while a marked quadratic effect of *X*_1_ was obtained; (c) When *X*_1_ was fixed, the contents of three macamides and two macaenes continuously increased until *X*_3_ reached approximately 30 min, and then decreased. In the same way, a variation of yield caused by *X*_3_ was also observed; (d) The function of *X*_2_ (temperature) and *X*_3_ (time) was studied when other variables were constant. The contents of three macamides and two macaenes constantly improved with the increase of both *X*_2_ and *X*_3_, and reached the maximum when *X*_2_ and *X*_3_ became approximately 40 °C and 30 min, respectively. Beyond this level, the yield reduced with the increase of *X*_2_ and *X*_3_. Thus, the interactive effect of *X*_2_ and *X*_3_ was significant; (e) The interactions between *X*_2_ (temperature) and *X*_4_ (power) was obvious. When *X*_2_ was set, the contents of three macamides and two macaenes improved with the increase of *X*_4_ and peaked at approximately 200 w, and then decreased. A same variation of yield caused by *X*_2_ was also observed; (f) When *X*_3_ (time) was fixed, the contents of three macamides and two macaenes showed a quadratic effect with the increase of *X*_4_ (power), while the yield was nearly unchanged at a fixed *X*_4_.

The final optimal extraction conditions were determined as follows: the ratio of solution to solid of 8.45:1 (mL/g), the extraction temperature of 37.7 °C, the extraction time of 27.8 min, and the extraction power of 208 W. To verify the accuracy of the response model, verification experiments were performed under optimum conditions: the ratio of solution to solid of 10:1 (mL/g), the extraction temperature of 40 °C, the extraction time of 30 min, and the extraction power of 200 W. The experimental yield was 1175.18 μg/g, which were close to the predicted yield of 1178.09 μg/g (relative error 0.25%). The above data indicated the effectiveness of macamides and macaenes extraction using UAE.

### 2.3. Qualitative Analysis

Over thirteen peaks were detected within 20 min in the mass spectrometry total ion current (TIC) chromatograms obtained in positive and negative modes. The TIC chromatograms of the reference standards and extracts of Maca are shown in Figure S1 (Supplementary Material). The molecular ion peaks in the mass spectra and comparative retention times for eleven macamides and two macaenes detected in the extracts were identical to those reference standards (Table 3) and the chemical profile reports of *Lepidium meyenii* Walp [1]. In this study, the macamides were sensitive in the positive mode, but the macaenes had higher sensitivity in the negative mode. The main fragment ion peaks detected from the macamides via MS/MS analysis were *m*/*z* 91.05 and *m*/*z* 121.06, corresponding to the benzyl (C_7_H_7_^+^) and methoxybenzyl (C_8_H_9_O^+^) ions, respectively. This was also previously reported [7,11].

### 2.4. Quantitative Analysis

**Figure 3:**
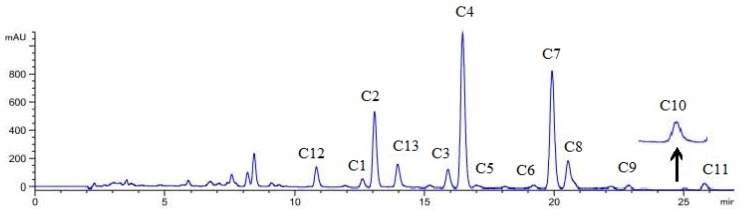
HPLC chromatography of macamides and macaenes from Maca hypocotyls. (C1: *N*-(3-methoxybenzyl)-linolenicamide, C2: *N*-benzyl-(9*Z*,12*Z*,15*Z*)-octadecatrienamide, C3: *N*-(3-methoxybenzyl)-(9*Z*,12*Z*)-octadecadienamide, C4: *N*-benzyl-(9*Z*,12*Z*)-octadecadienamide, C5: *N*-benzylpentadecanamide, C6: *N*-(3-methoxybenzyl)hexadecanamide, C7: *N*-benzylhexadecanamide, C8: *N*-benzyl-(9*Z*)-octadecenamide, C9: *N*-benzylheptadecanamide, C10: *N*-(3-methoxybenzyl)-octadecanamide, C11: *N*-benzyloctadecanamide, C12: 9*E*,12*E*,15*E*-octadecadienoic acid, C13: 9*E*,12*E*-octadecadienoic acid).

A developed HPLC method was used to determine the contents of specific macamides and macaenes in Maca hypocotyls from twenty different areas of Tibet (Table S1). The results are shown in Table 4 and Table 5 and the method was fully validated. Due to the fact peak C8 included two isomers, and baseline separation (*R* = 0.4) was not achieved by the HPLC method used (Figure 3), its content was not analyzed. A similar phenomenon had been reported in a previous article [24]. Linear regression equations, correlation coefficients (*R*^2^), and ranges of calibration curves for the listed compounds are shown in Table S2. All calibration curves showed good linear regression (*R*^2^ > 0.9990) within the test ranges. The LODs (limit of detection, S/N = 3) and LOQs (limit of quantification, S/N = 10) for the twelve investigated compounds were less than 20.21 ng and 59.73 ng, respectively (Table S2). The overall intra- and inter-day variations were within 0.68–2.66% and 0.66–2.50% for the twelve analytes. Validation studies of this method showed a good repeatability with RSD less than 3.0% (*n* = 3) for the investigated analytes (Table S3). As shown in Table S4, the developed analytical method had an excellent accuracy, with an overall recovery from 96.50 to 101.80% (*n* = 3) for the analytes. All the above indicates that this HPLC method was precise, accurate and sensitive enough for the simultaneous quantitative evaluation of the twelve main macamides and macaenes in Maca hypocotyls.

### 2.5. Principal Component Analysis (PCA) of the Samples

**Figure 4:**
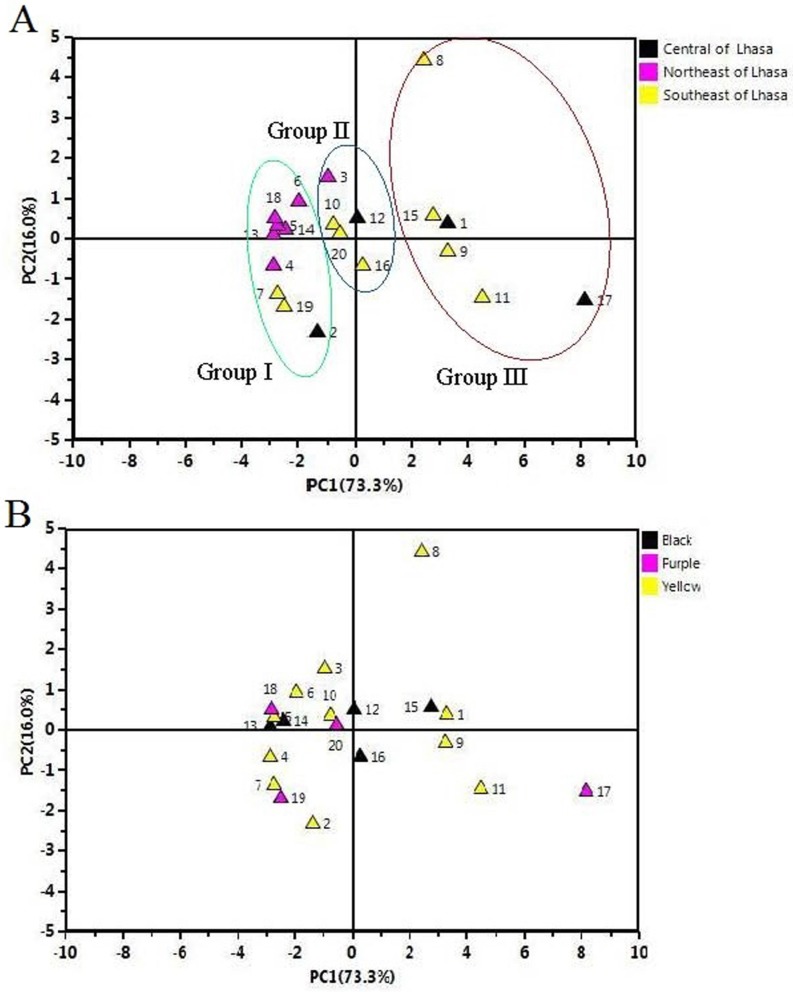
The principal component analysis (PCA) of black/purple/yellow Maca from different geographical origins.

The contents of twelve main macamides and macaenes were subjected to PCA to differentiate the cultivation areas and ecotypes of Maca hypocotyls. The results are shown in Figure 4. The first principal component (PC1) contains the most variance in the data and the second principal component (PC2) represents the maximum amount of variance not explained by PC1. The two ranking PCs, PC1 and PC2, described 73.3% and 16.0% of the total variability in the original observations, and consequently all the PCs accounts for 89.3% of the total variance. PC1 was the main variance factor. 

**Figure 5:**
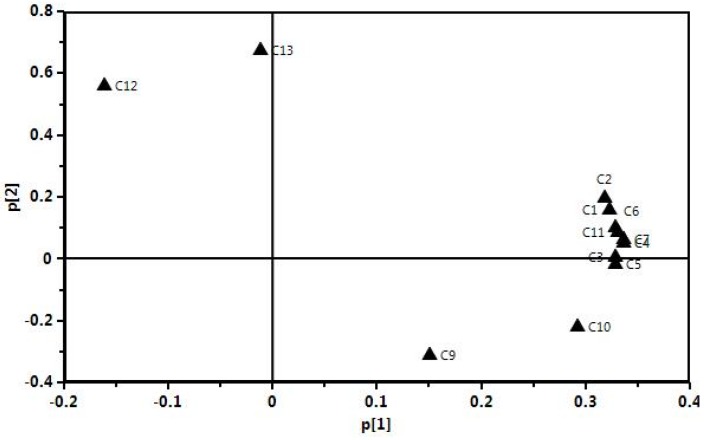
The loading plots of PC1 versus PC2 for twelve compounds in their profiles of twenty Maca hypocotyls samples.

The scores plots for PC1 versus PC2 (Figure 4A) showed the differences between these samples. The scores plot (Figure 4A) showed that twenty samples of Maca hypocotyls were clarified into three groups (Groups I–III) according to PC1. Group III was clustered by positive values of PC1, Group II was clustered in the middle according to PC1, while Group I was clustered by negative values of PC1. The total contents of twelve main macamides and macaenes in Group III that cultivated in the Southeast and Central of Lhasa were much higher than others (4.38 mg/g). In contrast, the total contents in Group I which was gathered from the most samples cultivated in the Northeast of Lhasa were much lower, and were no more than 1.23 mg/g, so the geographical origin played a more important role in the content of macamides. In addition, the differences in content of macamides caused by the variance in colours were not obvious (Figure 4B), and the different colours of Maca clustered together. This result was identical to the previous reported [7,25]. The loading plots for PC1 versus PC2 are shown in Figure 5. 

**Figure 6:**
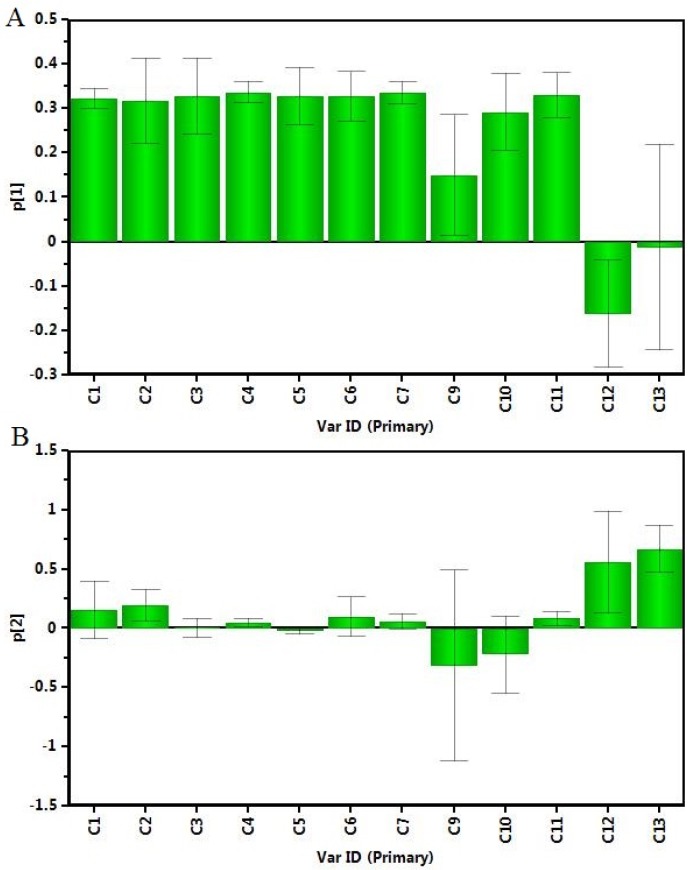
The influences of each variable on (**A**) the first component, (**B**) the second component.

A more detailed interpretation of the loadings can be done from plots showing the loadings separately (shown in Figure 6). In Figure 6A–B, the influence of each variable (C1–C13) on the two components was observed, C1–C11 mainly affected PC1, while C12 and C13 mainly affected PC2.

## 3. Materials and Methods

### 3.1. Plant Materials

Twenty Maca hypocotyls samples (fresh, 2 kg each) were collected from different cultivation areas of Tibet (China), in December 2016. Then they were dried at 40 °C for 24 h in a vacuum oven [15]. Maca hypocotyls were ground to powder (40-mesh) using an electrical JP-1000C-8 mill (Yongkang Instrument Co., Ltd., Yongkang, China) and the powder stored at 4 °C until use.

### 3.2. Chemicals and Reagents

Anhydrous ethanol, methanol, petroleum ether, cyclohexane and other chemicals were all analytical grade and got from Tianjin Chemical Reagent Co., Ltd. (Tianjin, China). HPLC grade acetonitrile was acquired from Sigma–Aldrich Chemical Co. (St. Louis, MO, USA). The macamides and macaenes standards were obtained from Wuhan Huaster Industrial Biotechnology Development Co., Ltd. (Wuhan, China). All standards were of purity greater than 98%.

### 3.3. Equipment

The extraction procedure was conducted in an ultrasound bath (SB-5200 DTD, frequency 40 kHz, maximum to 300 W (Ningbo Scientz Biotechnology Co., Ltd., Ningbo, China). The temperature was controlled within ±0.5 °C with a calibrated thermometer and adjusted with cold water. The extract was concentrated under vacuum by a EYELA N-1100 evaporator (Tokyo Rikakikai Co., Ltd., Tokyo, Japan).

### 3.4. Ultrasound-Assisted Extraction

Ground Maca hypocotyls powder (5.0 g) were transferred to 250 mL glass tubes with screw caps and the extraction procedure was conducted, according to the different conditions under study. Each extraction was repeated additional time. Then the extracts were filtrated and combined together to remove the solvents under vacuum at 40 °C. The obtained residue was dissolved in 10 mL methanol. These solutions were filtered through a 0.22 μm syringe filter and kept at 4 °C prior to qualitative and quantitative analysis.

### 3.5. Single Factor Experiment

Solvent was one of the most important factors affecting the extraction efficiency of bioactive compounds from plant materials. Macamides and macaenes were a group of non-polar compounds, previous studies have used petroleum ether [1,2,11], n-hexane [5,16], ethanol [8,9] and methanol [26] as the solvents for extracting them from Maca hypocotyls for further analysis. In this study, five different solvents anhydrous ethanol, methanol, ethyl acetate, petroleum ether and cyclohexane were selected to compare the efficiency of the extraction by UAE (ratio of solution to solid: 10:1 (mL/g), temperature: 40 °C, time: 30 min, power: 200 W). Ratio of solution to solid was a crucial parameter to improve the extraction yield and reduce the waste of solvent, so a series of ratios (mL/g) (5:1, 10:1, 15:1, 20:1, and 25:1) were investigated in this study. In addition, during the UAE progress, the extraction temperature, extraction power, extraction time, and extraction frequency were also the main factors that affect the extraction efficiency [27,28,29].

Hence, the effects of the extraction solvent, ratio of solution to solid, extraction temperature, extraction power, extraction time, and extraction frequency were evaluated by a single-factor design. Each experiment was carried out with 5.0 g of Maca hypocotyls powder, and the effects of each factor were investigated by analyzing the content of typical three macamides and two macaenes (Figure 1). The detailed conditions for each test were as follows: (a) sample was mixed with 50 mL anhydrous ethanol, methanol, ethyl acetate, petroleum ether and cyclohexane, respectively, and extraction test was performed at 40 °C and 200 W for 30 min; (b) when extraction frequency were at 1, 2, and 3, sample was extracted with 50 mL petroleum ether at 40 °C and 200 W for 30 min; (c) sample was mixed with 25, 50, 75, 100, and 125 mL petroleum ether to produce the corresponding solvent to-solid ratios of 5, 10, 15, 20, 25 mL/g, the extraction test was performed at 40 °C and 200 W for 30 min; (d) when extraction time was at 10, 20, 30, 40, and 50 min, sample was mixed with 50 mL petroleum ether at 40 °C and 200 W; (e) when extraction temperatures were at 20, 30, 40, 50, and 60 °C, sample was extracted with 50 mL petroleum ether at 200 W for 30 min; (f) when ultrasonic powers were at 100, 150, 200, 250, and 300 W, sample was extracted with 50 mL petroleum ether at 40 °C for 30 min.

### 3.6. Experimental Design and Data Dnalysis

RSM has been widely used in the extraction process and functional foods research as an effective statistical model [12,21,29]. A Box–Behnken design (BBD) with four independent variables was used in this research: ratio of solution to solid (*X*_1_), extraction temperature (*X*_2_), extraction time (*X*_3_), and extraction power (*X*_4_), and each variable was investigated at three levels (−1, 0, 1). The variables ranges were determined by the preliminary single factor test. The four independent variables resulted in an experimental design of twenty-nine experiments (Table 2).

Experimental data were fitted to a second-order polynomial model and regression coefficients obtained. The generalized second-order polynomial model used in the response surface analysis was as following Equation (2):

(2) $$Y=\beta_{0}+\sum_{i=1}^{4}\beta_{i}X_{i}+\sum_{i=1}^{4}\beta_{ii}X_{i}^{2}+\sum_{i=1}^{3}\sum_{j=i+1}^{4}\beta_{ij}X_{i}X_{j}$$

where *Y* represents the dependent variable; *β*_0_ is the constant coefficient; *β_i_*, *β_ii_* and *β_ij_* represent the model coefficients of the linear, quadratic and interaction effects of the variables, respectively; *X_i_* and *X_j_* are the coded independent variables.

Analysis of the experimental design and data were carried out using Design-Expert (version 8.0, StatEase Inc., Minneapolis, MN, USA). The statistical significance of the equation was examined by the analysis of variance (ANOVA). The significance of each coefficient and the interaction between each independent variable were evaluated according to the *P*-value.

### 3.7. Chemical Characterization of Extracts Obtained at Optimized Conditions

#### 3.7.1. Qualitative Analysis

In qualitative analysis, the assay was performed on an Agilent 1290 Infinity Liquid Chromatography system (Agilent Technologies, Burlington, MA, USA), equipped with a quaternary pump, an online vacuum degasser, an autosampler and a thermostatic column compartment was used to perform the separation of the multicomponents. Desirable chromatographic separation of macamides and macaenes in Maca hypocotyls was obtained on an Agilent ZORBAX RRHD Eclipse Plus C_18_ column (100 mm × 2.1 mm id, 1.8 μm) connected with a Phenomenex Security Guard ULTRA Cartridge (UHPLC C18, 2.1 mm id) using mobile phase A (0.1% formic acid aqueous solution) and mobile phase B (acetonitrile) in a gradient elution program: 0→20 min, 60→85% B. The flow rate was 0.5 mL/min. The wavelength was set at 210 nm and the temperature was set at 40 °C. The inject volume was 0.5 μL. The high accuracy mass spectrometric data were recorded on an Agilent QTOF 6550 mass spectrometer (Agilent, Waldbronn, Germany) equipped with an ESI source with Agilent Jet Steam (AJS) technology in positive ion mode. The optimized parameters were obtained as follows: gas temperature: 250 °C, gas flow: 5 L/min, nebulizer: 20 psi, sheath gas temperature: 350 °C, sheath gas flow: 11 L/min, capillary voltage: 4000 V, nozzle voltage: 500 V, fragmentor: 365 V, collision energy: 20 eV. The mass spectrometer was in full scan ranges of *m*/*z* 150−800 for MS and MS/MS. Data acquisition was controlled by the Agilent MassHunter Workstation Software (Version B.06.00, Agilent Technologies, Waldbronn, Germany).

In negative ion mode, the mobile phase A (water) and mobile phase B (acetonitrile) in a gradient elution program: 0→20 min, 65→100% B. The flow rate was 0.5 mL/min. The wavelength was set at 210 nm and the temperature was set at 40°C. The inject volume was 1 μL. The optimized parameters were obtained as follows: gas temperature: 250 °C, gas flow: 11 L/min, nebulizer: 45 psi, sheath gas temperature: 350 °C, sheath gas flow: 11 L/min, capillary voltage: 3500 V, nozzle voltage: 500 V, fragmentor: 365 V, OCT 1RF Vpp: 750 V, collision energy: 20 eV. The mass spectrometer was in full scan ranges of *m*/*z* 150-800 for MS and MS/MS.

#### 3.7.2. Quantitative Analysis

The quantitative analysis was performed on an Agilent 1260 high performance liquid chromatography (HPLC) system, equipped with a quaternary pump, an online vacuum degasser, UV-vis detector (DAD), manual sample injector and a Zorbax XDB C_18_ column (250 mm × 4.6 mm id; 5 µm). The solvent system consisted of water (A) and acetonitrile (B), using a gradient elution of 20:80 (*v*/*v*) (A:B) to 100 (B) in 30 min. The flow rate was set at 0.8 mL/min, and the column temperature was 40 °C. 10 µL sample was injected onto HPLC and monitored at 210 nm. Quantification of macamides and macaenes was done by the external standard method. The level of contents was expressed in μg/g dry weight. The method validation for quantitative analysis was fully conducted based on the linear regression, precision, repeatability, and recovery.

### 3.8. Statistical Analysis

All determinations were carried out in triplicate, and the experimental results obtained were expressed as mean values. The optimal extraction conditions were estimated through 3D RSM of two independent variables and each dependent variable. Statistical analysis and 3D graph were conducted using Design-Expert 8.0 Trial software (StatEase Inc., Minneapolis, MN, USA).

## 4. Conclusions

In this study, an efficient UAE method was established to extract macamides and macaenes from Maca hypocotyls. The optimal extraction conditions were determined to be the ratio of solution to solid of 10:1 (mL/g), the extraction temperature of 40 °C, the extraction time of 30 min, and the extraction power of 200 W by RSM method. Thus, UAE could be used to extract main active constituents from Maca hypocotyls for making use in healthcare and function food area. In addition, the differences of total contents of main macamides and macaenes between twenty different cultivated areas with three ecotypes showed that geographical origin played a more important role than colour.

## Figures and Tables

**Table 1:** Box-Behnken Design for independent variables and observed responses.

Run No.	*X*_1_: Ratio of Solution to Solid (mL/g)	*X*_2_ : Extraction Temperature (°C)	*X*_3_: Extraction Time (min)	*X*_4_: Extraction Power (W)	Content (μg/g)
1	0 (10:1)	0 (40)	0 (30)	0 (200)	1173.80
2	0 (10:1)	−1 (30)	0 (30)	–1 (150)	1017.79
3	0 (10:1)	0 (40)	0 (30)	0 (200)	1186.45
4	–1 (5:1)	0 (40)	0 (30)	1 (250)	1076.28
5	0 (10:1)	–1 (30)	0 (30)	1 (250)	1047.24
6	0 (10:1)	0 (40)	–1 (20)	1 (250)	1086.08
7	0 (10:1)	0 (40)	0 (30)	0 (200)	1158.12
8	0 (10:1)	0 (40)	0 (30)	0 (200)	1164.44
9	0 (10:1)	1 (50)	1 (40)	0 (200)	1038.88
10	0 (10:1)	1 (50)	0 (30)	1 (250)	985.32
11	1 (15:1)	0 (40)	1 (40)	0 (200)	1032.04
12	0 (10:1)	0 (40)	1 (40)	–1 (150)	1046.16
13	–1 (5:1)	0 (40)	0 (30)	–1 (150)	970.92
14	–1 (5:1)	1 (50)	0 (30)	0 (200)	1044.56
15	0 (10:1)	1 (50)	0 (30)	–1 (150)	988.48
16	1 (15:1)	1 (50)	0 (30)	0 (200)	1001.09
17	–1 (5:1)	–1 (30)	0 (30)	0 (200)	1099.08
18	0 (10:1)	0 (40)	1 (40)	1 (250)	1047.16
19	1 (15:1)	0 (40)	–1 (20)	0 (200)	1002.16
20	0 (10:1)	0 (40)	–1 (20)	–1 (150)	1036.44
21	0 (10:1)	1 (50)	–1 (20)	0 (200)	1000.48
22	1 (15:1)	0 (40)	0 (30)	–1 (150)	1010.80
23	0 (10:1)	–1 (30)	1 (40)	0 (200)	1036.68
24	1 (15:1)	–1 (30)	0 (30)	0 (200)	1041.04
25	0 (10:1)	–1 (30)	–1 (20)	0 (200)	1108.80
26	1 (15:1)	0 (40)	0 (30)	1 (250)	966.24
27	0 (10:1)	0 (40)	0 (30)	0 (200)	1163.08
28	–1 (5:1)	0 (40)	–1 (20)	0 (200)	1124.24
29	–1 (5:1)	0 (40)	1 (40)	0 (200)	1095.32

**Table 2:** ANOVA of response surface quadratic model analysis for the extraction yield.

Source	Sum of Squares	DF	Mean Square	*F*-Value	*P*-Value
Model	1.02 × 10^5^	14	7.91 × 10^3^	22.28	<0.0001 ***
*X*_1_	1.06 × 10^4^	1	1.06 × 10^4^	29.92	<0.0001 ***
*X*_2_	7.10 × 10^3^	1	7.10 × 10^3^	19.99	0.0005 **
*X*_3_	3.20 × 10^2^	1	3.20 × 10^2^	0.90	0.3586
*X*_4_	1.58 × 10^3^	1	1.58 × 10^3^	4.45	0.0533
*X*_1_*X*_2_	53.12	1	5.3.12	0.15	0.7049
*X*_1_*X*_3_	8.64 × 10^2^	1	8.64 × 10^2^	2.43	0.1410
*X*_1_*X*_4_	5.62 × 10^3^	1	5.62 × 10^3^	15.82	0.0014 **
*X*_2_*X*_3_	3.05 × 10^3^	1	3.05 × 10^3^	8.60	0.0109 *
*X*_2_*X*_4_	2.66 × 10^2^	1	2.66 × 10^2^	0.75	0.4015
*X*_3_*X*_4_	5.91 × 10^2^	1	5.91 × 10^2^	1.67	0.2177
*X*_1_^2^	2.68 × 10^4^	1	2.68 × 10^4^	75.42	<0.0001 ***
*X*_2_^2^	3.27 × 10^4^	1	3.27 × 10^4^	92.21	<0.0001 ***
*X*_3_^2^	1.06 × 10^4^	1	1.06 × 10^4^	29.85	<0.0001 ***
*X*_4_^2^	4.95 × 10^4^	1	4.95 × 10^4^	139.42	<0.0001 ***
Residual	4.97 × 10^3^	14	3.55 × 10^2^		
Lack of Fit	4.47 × 10^3^	10	4.47 × 10^2^	3.56	0.1160
Pure Error	5.02 × 10^2^	4	1.25 × 10^2^		
Cor Total	1.16 × 10^5^	28			

* Means significant at *P* < 0.05, ** means significant at *P* < 0.01, *** means significant at *P* < 0.0001.

**Table 3:** Chemical profiling of macamides and macaenes identified by UHPLC-EIS-Q-TOF-MS/MS analysis of extracts of the Maca hypocotyls.

Compounds	Retention Time (min)	Chemical Structure	Molecular Formula	Measured Value (*m/z*)	Main Fragment Ions
C1	6.388	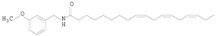	C_26_H_39_NO_2_	398.3073 [M + H]^+^	138.0920,121.0653
C2	6.643	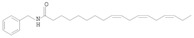	C_25_H_37_NO	368.2968 [M + H]^+^	108.0812, 91.0549
C3	9.006	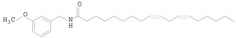	C_26_H_41_NO_2_	400.3227 [M + H]^+^	121.0645
C4	9.316	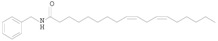	C_25_H_39_NO	370.3127 [M + H]^+^	91.0547
C5	9.912	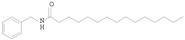	C_22_H_37_NO	332.2953 [M + H]^+^	91.0551
C6	11.061	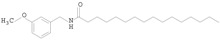	C_24_H_41_NO_2_	376.3225 [M + H]^+^	138.0918, 121.0652
C7	11.447	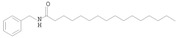	C_23_H_39_NO	346.3125 [M + H]^+^	91.0548
C8	13.005	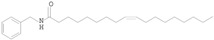	C_25_H_41_NO	372.3276 [M + H]^+^	108.0808, 91.0547
C9	14.507	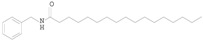	C_24_H_41_NO	360.3282 [M + H]^+^	91.0548
C10	17.335	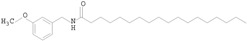	C_26_H_45_NO_2_	404.3546 [M + H]^+^	138.0915, 121.0653
C11	17.876	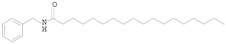	C_25_H_43_NO	374.3437 [M + H]^+^	91.0549
C12	2.673	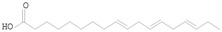	C_18_H_30_O_2_	277.2173 [M − H]^−^	
C13	3.91	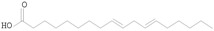	C_18_H_32_O_2_	279.2334 [M − H]^−^	

**Table 4:** The amounts of ten main macamides and two main macaenes in yellow Maca hypocotyls of different areas of Tibet.

Analytes	Content (μg/g)										
	Yellow Maca										
	1	2	3	4	5	6	7	8	9	10	11
C1	48.37 ± 0.66	12.03 ± 0.26	20.13 ± 0.83	2.09 ± 0.17	5.25 ± 0.44	11.77 ± 0.47	3.36 ± 0.25	65.93 ± 0.70	50.48 ± 0.60	13.48 ± 0.73	49.78 ± 0.45
C2	319.54 ± 5.65	71.55 ± 2.33	179.46 ± 5.20	4.07 ± 0.14	27.13 ± 0.55	80.34 ± 2.80	11.74 ± 0.25	520.73 ± 0.45	306.60 ± 4.76	158.87 ± 4.67	484.85 ± 4.51
C3	77.37 ± 1.41	9.79 ± 0.16	15.39 ± 0.66	1.19 ± 0.03	3.08 ± 0.12	11.80 ± 0.45	2.24 ± 0.08	55.75 ± 1.43	68.52 ± 1.43	15.27 ± 0.31	63.85 ± 1.21
C4	792.77 ± 10.44	95.80 ± 4.17	200.59 ± 6.53	15.49 ± 0.14	40.97 ± 0.27	137.33 ± 3.61	27.20 ± 0.52	693.99 ± 3.36	628.00 ± 9.96	285.06 ± 13.85	951.90 ± 9.73
C5	23.01 ± 0.57	6.97 ± 0.19	3.12 ± 0.11	-	-	-	-	13.79 ± 0.42	21.54 ± 1.00	5.33 ± 0.17	17.82 ± 0.14
C6	58.64 ± 1.27	-	28.48 ± 0.56	-	-	12.81 ± 0.21	-	50.15 ± 1.09	66.17 ± 1.40	39.39 ± 1.05	66.34 ± 0.56
C7	935.14 ± 9.48	91.79 ± 0.44	266.04 ± 5.52	10.33 ± 0.20	26.13 ± 0.58	80.65 ± 2.30	20.81 ± 0.30	776.32 ± 12.46	880.22 ± 21.17	346.25 ± 6.39	1018.49 ± 8.52
C9	20.52 ± 0.62	62.31 ± 2.36	6.45 ± 0.28	0.89 ± 0.02	3.20 ± 0.11	2.85 ± 0.11	1.80 ± 0.05	9.84 ± 0.22	19.50 ± 0.45	4.72 ± 0.14	14.12 ± 0.20
C10	4.19 ± 0.21	-	-	-	-	-	-	-	10.80 ± 0.30	-	21.82 ± 0.99
C11	70.02 ± 0.86	15.54 ± 0.59	34.48 ± 1.71	-	-	18.66 ± 0.89	-	58.19 ± 0.52	70.96 ± 2.53	21.79 ± 0.17	78.99 ± 2.80
C12	308.31 ± 5.96	380.98 ± 11.13	653.97 ± 16.75	473.95 ± 13.96	571.64 ± 2.87	529.31 ± 16.42	341.36 ± 1.85	875.03 ± 24.73	325.54 ± 6.18	420.43 ± 12.48	220.65 ± 2.87
C13	330.89 ± 13.42	197.32 ± 6.71	340.79 ± 5.30	216.99 ± 3.37	286.53 ± 10.33	346.17 ± 10.77	193.73 ± 2.82	476.05 ± 11.29	272.68 ± 4.82	304.74 ± 9.04	185.17 ± 2.84
Total	2988.77 ± 50.55	944.08 ± 28.34	1748.90 ± 43.45	725.00 ± 18.02	963.93 ± 15.27	1231.69 ± 35.73	602.24 ± 6.12	3595.77 ± 56.67	2721.01 ± 60.60	1615.33 ± 49.00	3173.78 ± 34.82

**Table 5:** The amounts of ten main macamides and two main macaenes in black and purple Maca hypocotyls of different areas of Tibet.

Analytes	Content (μg/g)								
	Black Maca					Purple Maca			
	12	13	14	15	16	17	18	19	20
C1	21.84 ± 0.99	2.63 ± 0.18	10.24 ± 0.33	32.11 ± 0.54	29.71 ± 0.24	81.74 ± 0.89	3.92 ± 0.13	5.36 ± 0.39	13.33 ± 0.55
C2	174.65 ± 4.42	3.44 ± 0.08	53.37 ± 1.83	367.70 ± 7.06	219.94 ± 3.61	539.51 ± 6.30	13.97 ± 0.53	19.80 ± 0.29	145.42 ± 1.37
C3	36.09 ± 0.84	0.87 ± 0.03	6.69 ± 0.21	36.03 ± 0.57	42.44 ± 1.73	143.13 ± 1.34	2.84 ± 0.11	4.13 ± 0.03	14.58 ± 0.61
C4	444.00 ± 8.57	13.66 ± 0.57	69.51 ± 2.32	624.32 ± 10.70	343.07 ± 6.77	1383.67 ± 8.02	31.22 ± 1.42	38.18 ± 0.64	252.42 ± 0.47
C5	10.84 ± 0.17	-	-	26.22 ± 0.52	7.50 ± 0.47	42.18 ± 0.46	-	-	10.44 ± 0.41
C6	30.97 ± 1.35	-	-	61.79 ± 1.62	35.09 ± 0.92	96.22 ± 4.01	-	-	34.60 ± 1.52
C7	417.42 ± 8.67	9.81 ± 0.41	57.20 ± 0.80	1023.70 ± 20.95	383.68 ± 6.77	1571.22 ± 6.57	16.71 ± 0.44	29.05 ± 0.07	401.57 ± 8.72
C9	12.53 ± 0.17	1.94 ± 0.07	3.62 ± 0.17	11.46 ± 0.31	6.95 ± 0.17	38.03 ± 0.64	3.61 ± 0.06	18.54 ± 0.13	6.63 ± 0.20
C10	2.72 ± 0.13	-	-	6.99 ± 0.16	6.71 ± 0.31	19.71 ± 0.64	-	-	-
C11	19.33 ± 0.20	-	10.15 ± 0.21	69.58 ± 1.07	21.46 ± 0.44	104.13 ± 2.10	-	-	34.23 ± 1.54
C12	366.72 ± 17.91	531.46 ± 20.04	563.16 ± 4.04	417.84 ± 12.20	329.02 ± 5.07	173.85 ± 1.74	600.06 ± 22.97	338.18 ± 2.90	412.03 ± 16.42
C13	365.56 ± 13.56	279.45 ± 7.35	269.40 ± 2.14	302.32 ± 7.46	244.40 ± 2.59	190.08 ± 8.40	298.12 ± 11.46	195.91 ± 2.05	290.77 ± 9.90
Total	1902.67 ± 56.98	843.26 ± 28.73	1043.34 ± 12.05	2980.06 ± 63.44	1669.97 ± 29.09	4383.40 ± 41.11	970.45 ± 37.12	649.15 ± 6.50	1616.02 ± 41.71

## Supplementary Materials

The Supplementary Materials are available online. Figure S1: The total ion current (TIC) chromatogram profiles of reference standards and the Maca hypocotyls sample. (A) Reference standards macamides in positive ion mode; (B) Reference standards macaenes in negative ion mode; (C) Maca hypocotyls sample in positive ion mode; (D) Maca hypocotyls sample in negative ion mode, Table S1: Samples of cultivated Maca from different Tibet areas, Table S2: Linear regression data, LOD, and LOQ of the investigated compounds, Table S3: Precision and repeatability of the investigated compounds, Table S4: Accuracy of HPLC method for the determination of investigated compounds.
