# Supplementary material for: Optimization of Ultrasound-Assisted Extraction, HPLC and UHPLC-ESI-Q-TOF-MS/MS Analysis of Main Macamides and Macaenes from Maca (Cultivars of Lepidium meyenii Walp)
Source: Molecules. 2017 Dec 10;22(12):2196. doi: 10.3390/molecules22122196 (PMC6149678; doi:10.3390/molecules22122196)
Supplement: Supplementary file 1 [file molecules-22-02196-s001.pdf]

Supplementary Material

Optimization of Ultrasound-Assisted Extraction, HPLC and UHPLC-ESI-Q-TOF-MS/MS Analysis of Main Macamides and Macaenes from Maca (Cultivars of *Lepidium Meyenii* Walp)

Shu-Xiao Chen [1], Ke-Ke Li [2,*], Duoji Pubu [3], Si-Ping Jiang [3], Bin Chen [4], Li-Rong Chen [1], Zhen Yang [5], Chao Ma [3], Xiao-Jie Gong [2,*]

1 College of Environmental and Chemical Engineering, Dalian University, Dalian 116622, China; 18340862946@163.com (S.-X.C); chenlirong@dlu.edu.cn (L.-R.C)
2 College of Medical, Dalian University, Dalian 116622, China;
3 Tibet Plateau Institute of Biology, Lhasa 850001, China; puduo@126.com (D.P); tpibjiangsp@126.com (S.-P.J); wvoo@163.com (C.M)
4 School of Marine Sciences, Sun Yat-sen University, Zhuhai 519082, China; chenyishan@126.com
5 HPV Immunology Lab, Leidos Biomedical Research Inc., Frederick National Laboratory for Cancer Research, Frederick, MD 21702, USA; zhenyang2@yahoo.com
* Correspondence: like905219@163.com (K.-K.L.); gxjclr@163.com (X.-J.G.); Tel.: +86-411-8740-3156 (K.-K.L. and X.-J.G.); Fax: +86-411-8733-0507 (K.-K.L. and X.-J.G.)

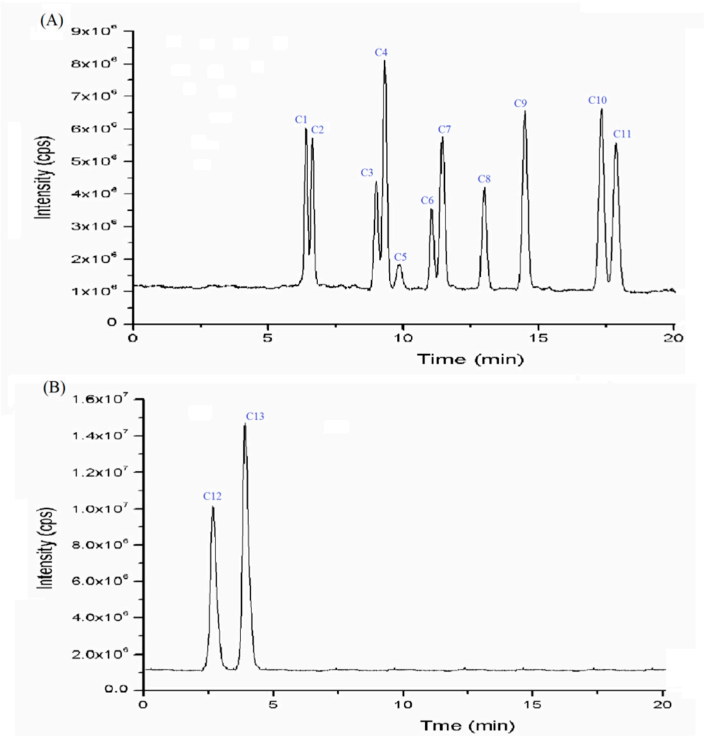

Figure S1. *Cont.*

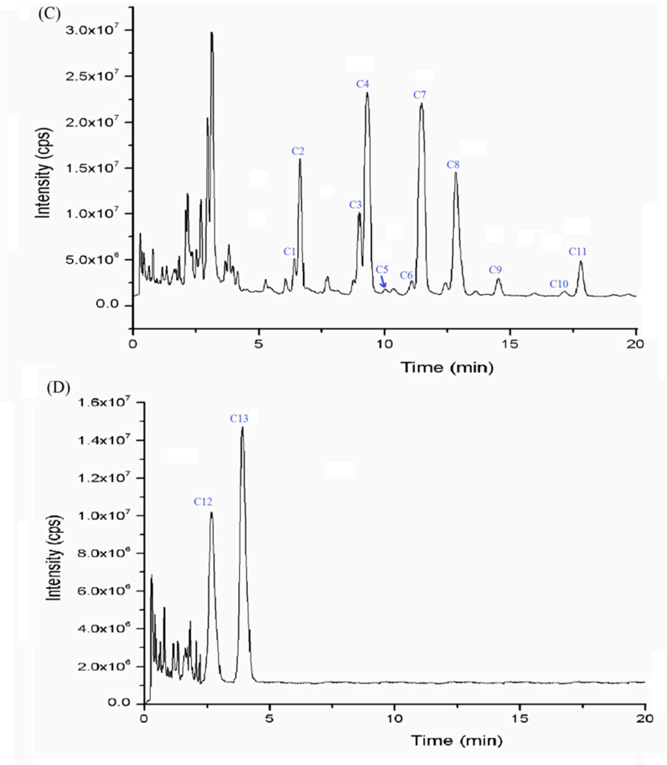

Figure S1. The total ion current (TIC) chromatogram profiles of reference standards and the Maca hypocotyls sample. (A) Reference standards macamides in positive ion mode; (B) Reference standards macaenes in negative ion mode; (C) Maca hypocotyls sample in positive ion mode; (D) Maca hypocotyls sample in negative ion mode. * C1: N-(3-methoxybenzyl)linolenicamide, C2: N-benzyl-(9*Z*,12*Z*,15*Z*)-octadecatrienamide, C3: N-(3-methoxybenzyl)-(9*Z*,12*Z*)-octadecadienamide, C4: N-benzyl-(9*Z*,12*Z*)-octadecadienamide, C5: N-benzylpentadecanamide, C6: N-(3-methoxybenzyl)hexadecanamide, C7: N-benzylhexadecanamide, C8: N-benzyl-(9*Z*)-octadecenamide, C9: N-benzylheptadecanamide, C10: N-(3-methoxybenzyl)-octadecanamide, C11: N-benzyl-octadecanamide, C12: 9*E*,12*E*,15*E*-octadeca- dienoic acid, C13: 9*E*,12*E*-octadecadienoic acid.

Table S1. Samples of cultivated Maca from different Tibet areas.

Ecotypes	No.	Samples
Yellow	1	Central Lhasa-1 (Lhasa city)
	2	Central Lhasa-2 (Lhasa city)
	3	Northeast of Lhasa-1 (Changdu city)
	4	Northeast of Lhasa-2 (Changdu city)
	5	Northeast of Lhasa-3 (Changdu city)
	6	Northeast of Lhasa-4 (Changdu city)
	7	Southeast of Lhasa-1 (Shannan city)
	8	Southeast of Lhasa-2 (Shannan city)
	9	Southeast of Lhasa-3 (Shannan city)
	10	Southeast of Lhasa-4 (Shannan city)
	11	Southeast of Lhasa-5 (Shannan city)

Table S1. *Cont*.

Black	12	Central Lhasa (Lhasa city)
	13	Northeast of Lhasa-1 (Changdu city)
	14	Northeast of Lhasa-2 (Changdu city)
	15	Southeast of Lhasa-1 (Shannan city)
	16	Southeast of Lhasa-2 (Shannan city)
Purple	17	Central of Lhasa (Lhasa city)
	18	Northeast of Lhasa (Changdu city)
	19	Southeast of Lhasa-1 (Shannan city)
	20	Southeast of Lhasa-2 (Shannan city)

Table S2. Linear regression data, LOD, and LOQ of the investigated compounds.

**Compounds**	**Regression equations**	**Linear ranges (μg/mL)**	R^2	**LOD (ng)**	**LOQ (ng)**
C1	y = 14.49x - 0.4902	1.3–82.57	0.9993	2.57	7.79
C2	y = 20.29x + 78.308	3.52–600	0.9994	1.46	4.38
C3	y = 19.989x + 11.205	0.84–200	0.9999	2.06	6.37
C4	y = 18.46x - 16.961	8–2000	0.9992	1.64	4.96
C5	y = 13.335x - 5.886	2–60	0.9994	10.35	31.06
C6	y = 7.2567x - 0.1765	10–100.76	0.9990	2.91	8.73
C7	y = 13.045x - 23.197	4–2000	0.9996	2.82	8.56
C9	y = 15.768x + 6.597	0.87–80	0.9995	7.85	23.64
C10	y = 12.629x + 0.3408	2.6–22.95	0.9995	4.93	9.86
C11	y = 11.428x - 14.514	10–120	0.9993	3.41	10.38
C12	y = 16.602x + 5.434	1.3–1000	0.9993	11.33	33.89
C13	y = 20.234x + 38.238	10–1000	0.9996	20.21	59.73

LOD, limit of detection; LOQ, limit of quantification.

Table S3. Precision and repeatability of the investigated compounds.

Compounds	Precision				Repeatability	
	Intra-day (n = 3)		Inter-day (n = 5)		Mean (μg/g)	RSD (%)
	Content (μg/mL)	RSD (%)	Content (μg/mL)	RSD (%)		
C1	50.04 ± 1.19	2.39	49.89 ± 0.93	1.86	32.11	1.68
C2	148.04 ± 2.08	1.41	148.75 ± 2.94	1.98	367.70	1.92
C3	200.09 ± 1.59	0.79	199.89 ± 1.33	0.66	36.03	1.58
C4	500.26 ± 2.75	0.55	500.45 ± 3.87	0.77	624.32	1.71
C5	20.35 ± 0.45	2.21	19.86 ± 0.32	1.61	26.22	1.98
C6	53.49 ± 1.24	2.32	53.26 ± 1.05	1.97	61.79	2.62
C7	501.24 ± 3.42	0.68	500.98 ± 3.95	0.79	1023.70	2.05
C9	25.42 ± 0.46	1.81	25.58 ± 0.64	2.50	11.46	2.71
C10	10.13 ± 0.27	2.66	10.07 ± 0.25	2.48	6.99	2.29
C11	56.48 ± 0.78	1.38	56.12 ± 0.64	1.14	69.58	1.54
C12	53.49 ± 1.24	2.32	53.26 ± 1.05	1.97	417.84	2.92
C13	54.71 ± 0.66	1.20	55.82 ± 0.60	1.08	302.32	2.47

RSD: relative standard deviation.

Table S4. Accuracy of HPLC method for the determination of investigated compounds.

Compounds	Original (μg)	Spiked (μg)	Found (μg)	Recovery (%)	RSD (%)
		40.00	61.26 ± 1.35	98.55	1.54
C1	21.84	20.00	41.94 ± 0.86	100.50	1.99
		10.00	31.57 ± 0..91	97.30	2.14
		350.00	524.94 ± 10.23	100.08	1.06
C2	174.65	150.00	324.12 ± 8.95	99.64	2.48
		80.00	254.58 ± 4.23	99.91	1.64
		60.00	96.22 ± 1.27	100.22	2.21
C3	36.09	30.00	66.15 ± 0.85	100.20	2.77
		15.00	51.36 ± 1.04	101.80	2.80
		800.00	1244.96 ± 8.37	100.12	1.69
C4	444.00	400.00	843.78 ± 6.23	99.95	1.60
		200.00	644.50 ± 8.51	100.25	2.24
		20.00	30.74 ± 0.45	99.50	1.83
C5	10.84	10.00	21.05 ± 0.56	102.10	2.53
		5.00	15.90 ± 0.38	101.20	1.65
		60.00	90.84 ± 2.12	99.78	0.65
C6	30.97	30.00	61.03 ± 1.62	100.20	0.95
		15.00	45.75 ± 0.78	98.53	1.68
		800.00	1217.26 ± 8.84	99.98	1.20
C7	417.42	400.00	817.05 ± 6.14	99.91	0.54
		200.00	617.33 ± 8.43	99.96	1.94
		20.00	32.66 ± 0.52	100.65	2.23
C9	12.53	10.00	22.48 ± 0.61	99.50	2.14
		5.00	17.56 ± 0.40	100.60	2.60
		4.00	6.69 ± 0.17	99.25	2.97
C10	2.72	2.00	4.65 ± 0.12	96.50	2.60
		1.00	3.70 ± 0.08	98.00	1.48
		40.00	59.46 ± 0.87	100.33	1.19
C11	19.33	20.00	39.27 ± 1.03	99.71	1.46
		10.00	29.34 ± 0.75	100.10	0.46
		600.00	965.90 ± 10.03	99.86	1.42
C12	366.72	300.00	666.87 ± 4.00	100.05	0.95
		150.00	516.73 ± 5.31	100.00	1.03
		600.00	965.67 ± 4.58	100.02	0.92
C13	365.56	300.00	665.51 ± 2.30	99.98	0.58
		150.00	515.74 ± 4.57	100.12	1.55
